# *Leptothrix ochracea* genomes reveal potential for mixotrophic growth on Fe(II) and organic carbon

**DOI:** 10.1128/aem.00599-24

**Published:** 2024-08-12

**Authors:** Gracee K. Tothero, Rene L. Hoover, Ibrahim F. Farag, Daniel I. Kaplan, Pamela Weisenhorn, David Emerson, Clara S. Chan

**Affiliations:** 1 Microbiology Graduate Program, University of Delaware, Newark, Delaware, USA; 2 Delaware Biotechnology Institute, Newark, Delaware, USA; 3 Department of Earth Sciences, University of Delaware, Newark, Delaware, USA; 4 School of Marine Science and Policy, University of Delaware, Newark, Delaware, USA; 5 Savannah River Ecology Laboratory, University of Georgia, Aiken, South Carolina, USA; 6 Argonne National Laboratory, Lemont, Illinois, USA; 7 Bigelow Laboratory for Ocean Sciences, East Boothbay, Maine, USA; Washington University in St. Louis, St. Louis, Missouri, USA

**Keywords:** *Leptothrix ochracea*, chemolithotrophy, mixotrophy, iron-oxidizing bacteria, iron microbial mats

## Abstract

*Leptothrix ochracea* creates distinctive iron-mineralized mats that carpet streams and wetlands. Easily recognized by its iron-mineralized sheaths, *L. ochracea* was one of the first microorganisms described in the 1800s. Yet it has never been isolated and does not have a complete genome sequence available, so key questions about its physiology remain unresolved. It is debated whether iron oxidation can be used for energy or growth and if *L. ochracea* is an autotroph, heterotroph, or mixotroph. To address these issues, we sampled *L. ochracea*-rich mats from three of its typical environments (a stream, wetlands, and a drainage channel) and reconstructed nine high-quality genomes of *L. ochracea* from metagenomes. These genomes contain iron oxidase genes *cyc2* and *mtoA,* showing that *L. ochracea* has the potential to conserve energy from iron oxidation. Sox genes confer potential to oxidize sulfur for energy. There are genes for both carbon fixation (RuBisCO) and utilization of sugars and organic acids (acetate, lactate, and formate). *In silico* stoichiometric metabolic models further demonstrated the potential for growth using sugars and organic acids. Metatranscriptomes showed a high expression of genes for iron oxidation; aerobic respiration; and utilization of lactate, acetate, and sugars, as well as RuBisCO, supporting mixotrophic growth in the environment. In summary, our results suggest that *L. ochracea* has substantial metabolic flexibility. It is adapted to iron-rich, organic carbon-containing wetland niches, where it can thrive as a mixotrophic iron oxidizer by utilizing both iron oxidation and organics for energy generation and both inorganic and organic carbon for cell and sheath production.

**IMPORTANCE:**

Winogradsky's observations of *L. ochracea* led him to propose autotrophic iron oxidation as a new microbial metabolism, following his work on autotrophic sulfur-oxidizers. While much culture-based research has ensued, isolation proved elusive, so most work on *L. ochracea* has been based in the environment and in microcosms. Meanwhile, the autotrophic *Gallionella* became the model for freshwater microbial iron oxidation, while heterotrophic and mixotrophic iron oxidation is not well-studied. Ecological studies have shown that *Leptothrix* overtakes *Gallionella* when dissolved organic carbon content increases, demonstrating distinct niches. This study presents the first near-complete genomes of *L. ochracea*, which share some features with autotrophic iron oxidizers, while also incorporating heterotrophic metabolisms. These genome, metabolic modeling, and transcriptome results give us a detailed metabolic picture of how the organism may combine lithoautotrophy with organoheterotrophy to promote Fe oxidation and C cycling and drive many biogeochemical processes resulting from microbial growth and iron oxyhydroxide formation in wetlands.

## INTRODUCTION

*Leptothrix ochracea* was one of the first described microorganisms (1–3), eminently recognizable both microscopically and to the naked eye, yet fundamental questions about its metabolism remain. It is a member of the *Leptothrix–Sphaerotilus* group within the Gammaproteobacteria, formerly known as the Betaproteobacteria, and its distinguishing characteristic is the prodigious production of iron-mineralized sheaths that are mostly devoid of cells. The cells produce sheaths rapidly (4), which become felted together into fluffy, orange, iron microbial mats in freshwater systems (3, 5–7). These sheaths represent a reservoir of mineral-associated organic carbon (8–11), which adsorb diverse heavy metals (e.g., U and actinides) and nutrients (e.g., P, N, S, and Si) (12–25). *L. ochracea* is primarily found in oxic freshwater streams, wetlands, and drainage channels with slowly flowing water, a source of ferrous iron, and low quantities of organic carbon. Winogradsky first hypothesized that *L. ochracea* grows as a chemolithoautotrophic iron oxidizer based on enrichments and environmental observations (3). He posited that its main energy source is ferrous iron based on observations that it requires iron for growth and produces a massive amount of iron oxides relative to a small cell yield. However, Winogradsky’s culture media contained organics from natural water, so neither a strictly autotrophic metabolism nor growth via iron oxidation was demonstrated. To this day, investigations have not confirmed the role of iron in *L. ochracea* metabolism (26–29).

Because *L. ochracea* has resisted isolation (30, 31), our understanding of its metabolism is based on enrichment or xenic cultures. Typical culturing methods have used organics, e.g. soil extract, and the repeated difficulty in demonstrating the role of iron oxidation has fueled the idea that *L. ochracea* is heterotrophic like characterized isolates of *Leptothrix* and *Sphaerotilus* (which also oxidize metals, but for unclear purposes). These isolates generally respond strongly to addition of organics, which is not true of *L. ochracea* (32), suggesting distinct differences in carbon metabolism and growth strategy. Recent laboratory-based studies on enrichment cultures of *L. ochracea* demonstrated that it can assimilate limited amounts of carbon from bicarbonate (4). Its enrichment from environmental samples required organic-containing environmental water and Fe(II), suggesting that it may use Fe(II) as an energy source (4). Furthermore, in an ecological time-series study, its abundance corresponded to higher quantities of dissolved organic carbon than the autotrophic iron oxidizers in the family Gallionellaceae (4, 32). Its association with higher organic carbon than Gallionellaceae and lower organic carbon than other *Leptothrix–Sphaerotilus* members gives it a distinct niche from these other metal-oxidizing bacteria.

Genomes can provide much insight into metabolic potential if they contain genes characteristic of specific energy and nutrient pathways. While there are genomes of other *Leptothrix* and *Sphaerotilus* isolates (29), those genomes are unlikely to be representative of *L. ochracea* because of its distinct responses to organic carbon. The first and only genome of *L. ochracea* was sequenced from a single cell, and as is typical of a single-cell amplified genome (SAG), the *L. ochracea* L12 SAG is only a partial genome, 0.51 Mb in size (4). Based on its tRNAs, the full genome size is roughly estimated to be 2.2 Mb (4), suggesting the majority of the genome is missing. Thus, the *L. ochracea* L12 SAG gives limited insights into the metabolism of *L. ochracea*. It contains genes for a cytochrome bd terminal oxidase, Form II RuBisCO, and electron transport, including alternative complex III, which could enable growth via chemolithoautotrophic iron oxidation. However, these components are not unique to iron oxidation, and the SAG lacks genes for known iron oxidases (e.g., *cyc2* and *mtoA*). The L12 SAG also contains genes for formate dehydrogenase, but lacks organic carbon utilization genes; therefore, whether it can gain energy or biomass from organics is unresolved. *L. ochracea*’s lifestyle suggests its role as a mixotrophic iron oxidizer, akin to an intermediate between the autotrophic iron-oxidizing Gallionellaceae and the heterotrophic metal-oxidizing *Leptothrix–Sphaerotilus* group. A complete genome would allow us to more thoroughly discern its metabolisms and evaluate its identity as an autotrophic, heterotrophic, or mixotrophic iron oxidizer.

To this end, we sequenced and analyzed the first near-complete *Leptothrix ochracea* genomes, reconstructed from metagenomes of freshwater iron mats sampled at the Savannah River Site (SRS) in South Carolina and Spruce Point in Boothbay Harbor, Maine. Nine high-quality, near-complete genomes were reconstructed from stream and wetland mats. These genomes demonstrate the potential for *L. ochracea* to grow as a mixotrophic iron oxidizer. It has the genomic potential to combine chemolithotrophy and organoheterotrophy to generate iron oxyhydroxides and biomass carbon. Metatranscriptomes and metabolic models of these genomes further support the role of *L. ochracea* as a mixotrophic iron oxidizer. Our results reveal metabolic connections between iron and carbon cycling within *L. ochracea*; these relationships help our understanding of the drivers of biogeochemical transformations catalyzed by iron-oxidizing microbes in wetlands.

## RESULTS AND DISCUSSION

### Iron microbial mat samples

**Fig 1:**
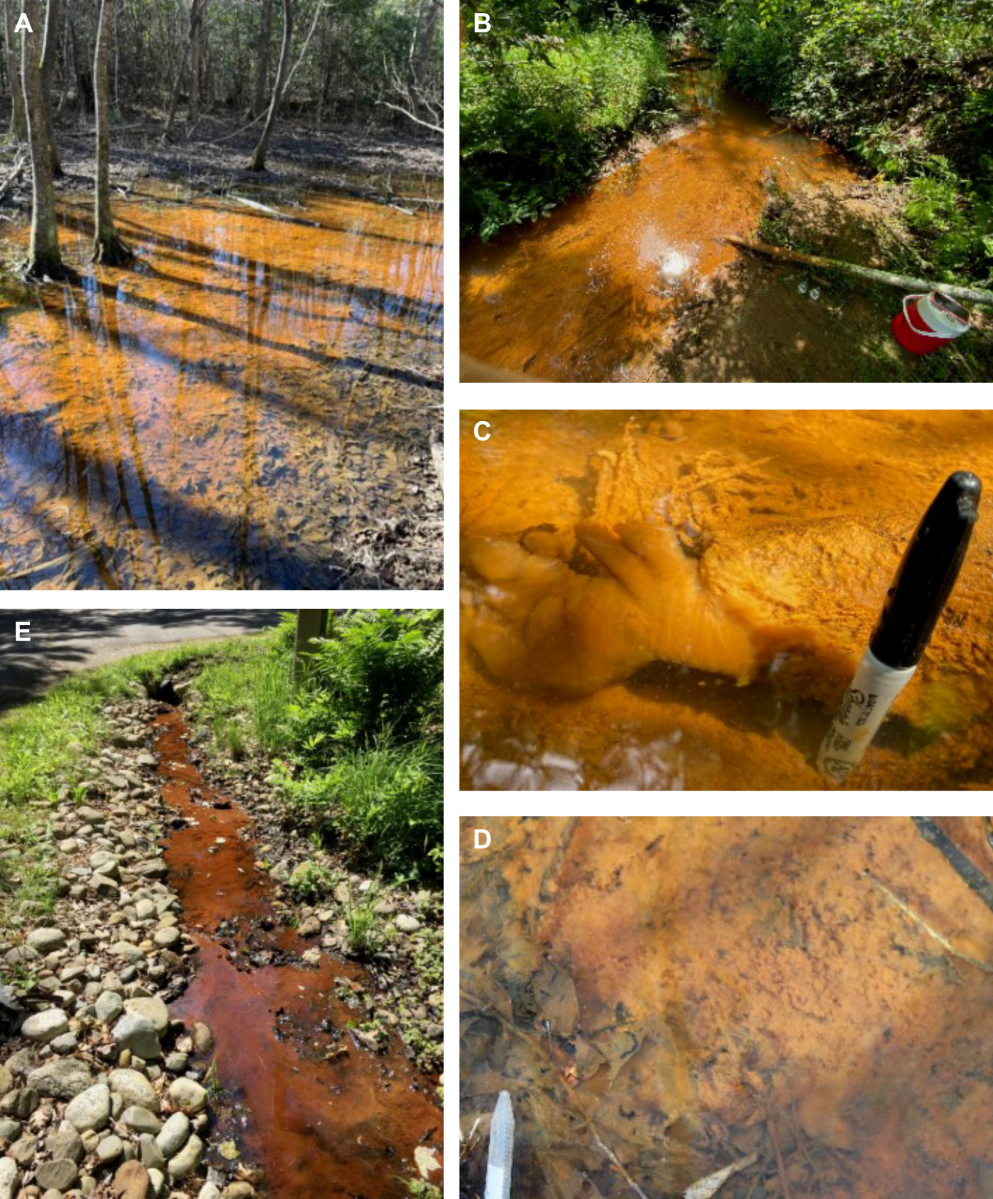
Iron microbial mat sampling locations. Images A–D are from the Savannah River Site; image E is from the Spruce Point site. (**A**) Extensive iron mat at the DE wetland site; (**B**) iron mat at the ISCO stream site; (**C**) fluffy *Leptothrix*-type iron mat at the ISCO site in the Tims Branch stream; (**D**) iron mat at the MN wetland site; (**E**) iron mat in the Spruce Point site drainage channel.

Sampling was performed in the Tims Branch stream and its surrounding wetlands at the Savannah River Site (SRS) in Jackson, SC, and at Spruce Point in Boothbay Harbor, ME. Across these two locations, four sites representing the typical environments for *L. ochracea* were sampled. These include two freshwater wetlands (SRS MN and DE sites), one freshwater stream (SRS ISCO site), and a drainage channel with slow-moving water (Spruce Point SP site) (Fig. 1). Mats sampled from the SRS wetland sites were dense, dark orange, and flocculent, whereas mats sampled from the SRS stream site and Spruce Point were orange, fluffy, and most prevalent around the stream/channel edges and in areas protected from high flow. In addition, a thin layer of dense, brown mat was observed at the SRS stream site on the surface of the sediment spanning the center of Tims Branch. The sampled mats had morphology matching the typical descriptions of *L. ochracea* mats (32–34).

*L. ochracea* mats are typically found within aerobic, circumneutral to slightly acidic freshwater environments with abundant Fe^2+^. To evaluate the geochemistry of these sampling sites, pH, oxygen, and Fe^2+^ were measured (Table 1). The surface water pH near these mats was circumneutral to slightly acidic. Oxygen concentrations ranged widely, with highest values in the ISCO stream site mats and at the surface of DE wetland mats. To evaluate oxygen gradients within these mats, dissolved oxygen measurements were taken near the mat surface and 1.5–2.5 cm below the mat surface. At the ISCO site, oxygen decreased inside the mats compared to the surface, yet mats remained aerobic. In contrast, mats at the MN and DE sites became anoxic at depth. The concentration of dissolved Fe^2+^ ranged from 1 to 79 µM in mat samples, though lower values may be due to mixing of surface water with mat samples. At the MN3 location, we used dialysis samplers to measure dissolved Fe^2+^ in sediment pore water directly below mats (Table S1). Here, Fe^2+^ was highest in sediment pore water near the mat/sediment interface (1250 µM, ~1 cm below mat surface), suggesting that there is a significant flux of ferrous groundwater into the surface water to support growth by iron oxidation.

**TABLE 1:** Geochemical parameters from the SRS and Spruce Point^*d*^

Sampling site	Sample	pH	O_2_ in surface water (µM)	O_2_ inside mat (µM)	[Fe^2+^] (µM)
SRS	ISCOA1K	6.1	153	109^*a*^	–
	ISCOB2	6.2	249	–	–
	ISCOC1	6.2	205	–	79.4
	MN2	–	–	–	33.3
	MN3	5.3	78.1	0.0^*b*^	8.02
	DE1^*c*^	5.5	219	0.0^*b*^	1.27
SP	4SP	6.1	20–80	–	9.50

^*a*^ Measurement was taken from 2.5 cm below the surface of the mat.

^*b*^ Measurement was taken from 1.5 cm below the surface of the mat.

^*c*^ Sample DE2 was a bulk sample taken over a large area, taken from the same location as site DE1. The geochemistry of DE2 is approximately the same as DE1.

^*d*^ –, not measured.

**Fig 2:**
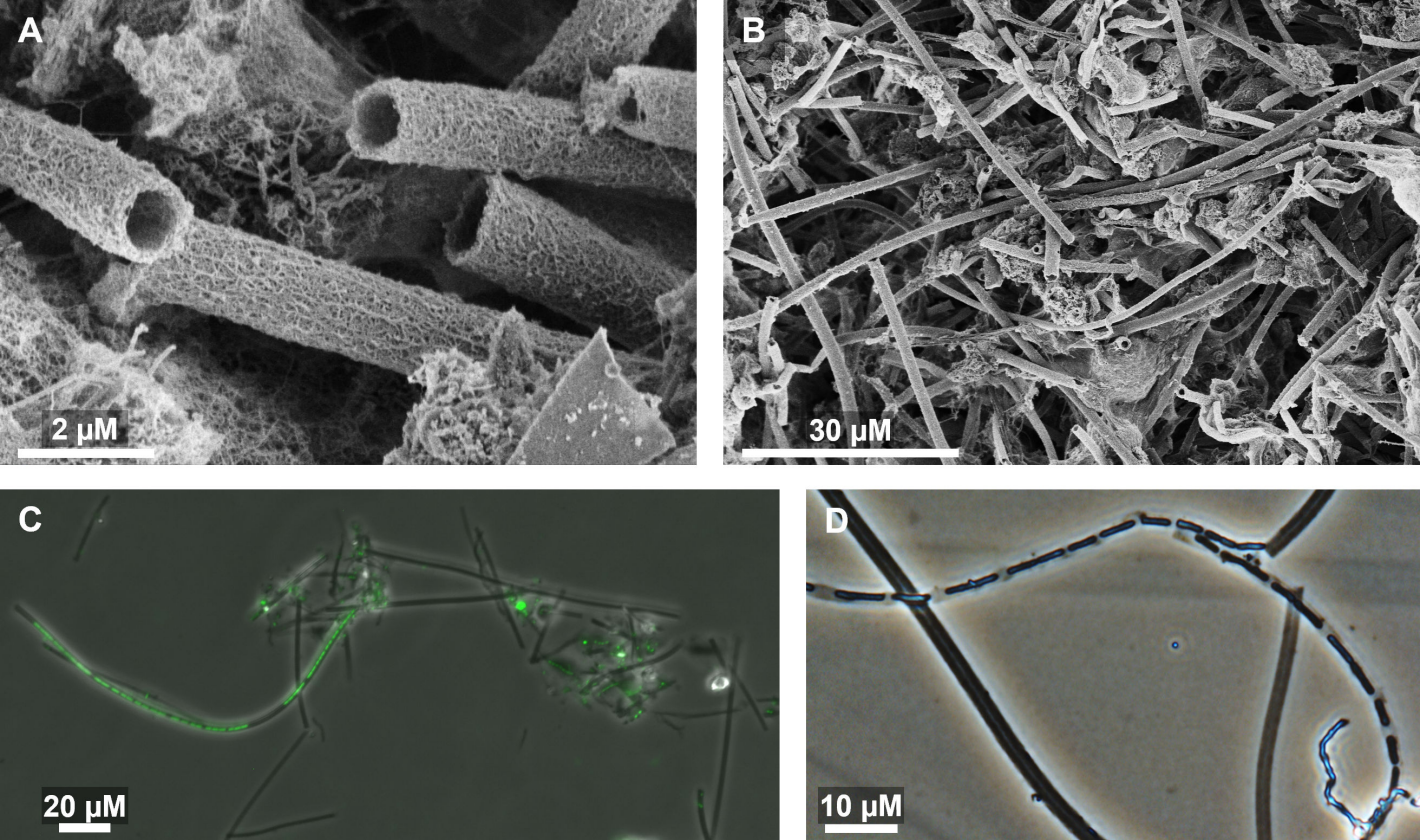
Microscopy of *Leptothrix* iron mats. (**A**) SEM of the *L. ochracea* sheath structure from the DE site mat; (**B**) SEM of the iron mat structure showing abundant *L. ochracea* sheaths in the DE site mat; (**C**) combined phase contrast and fluorescence microscopy of the mat from the DE site shows a chain of *L. ochracea* cells (visualized with Syto13 nucleic acid stain) within a sheath and several empty sheaths; (**D**) phase contrast light micrography of the mat from Spruce Point shows a chain of *L. ochracea* cells within a sheath and empty sheaths.

The SRS mats were characterized via microscopy to demonstrate the presence of sheaths typical of *L. ochracea*. Sheath-rich mats sampled intact from Spruce Point were previously characterized microscopically (34). This analysis (Fig. S1) and additional phase contrast imaging (Fig. 2D) showed that the Spruce Point mats were largely composed of empty sheaths, with the cells occupying sheath ends at the surface of the mat. The SRS mats also contained abundant sheaths characteristic of *Leptothrix ochracea*, some with chains of cells and others empty and abandoned by cells (Fig. 2). Such characteristics have previously been described in *Leptothrix ochracea* mats and are distinct from other *Leptothrix* and *Sphaerotilus* (5, 27). Both Spruce Point and SRS samples are consistent with typical morphological descriptions of *L. ochracea* and suggest that *L. ochracea* is a prominent and active iron oxidizer in these microbial mats. The massive production of empty mineralized sheath is analogous to the production of iron oxyhydroxide stalks by *Gallionella* and *Mariprofundus*. Sheath and stalk structures serve multiple purposes: (1) acting as a holdfast to position cells where they can access Fe(II), nutrients, and O_2_; (2) a repository for oxidized iron waste products; and (3) sheath production coupled to mineral deposition may assist and direct cell filament motility (34, 35). The extensive formation of Fe-mineralized sheaths suggests that Fe oxidation is an important part of *L. ochracea* metabolism and growth.

### Presence of *Leptothrix ochracea* 16S rRNA gene sequences

The microbial community composition of iron mat samples from the SRS was assessed by 16S rRNA gene analysis. After QC, there were 266,445 reads for eight samples (23,565–46,362 per sample), which clustered into 90,818 operational taxonomic units (OTUs, at 99% similarity) (Table S2). Based on initial SILVA classification, *Leptothrix* appeared to be either absent or at a very low level in these mats (0.01%–0.47%). However, we checked the SILVA classification of the *L. ochracea* sequences from the study by Fleming *et al*. (7) and found that they are incorrectly classified as the genus *Paucibacter*. Three abundant (>0.5%) OTUs classified as *Paucibacter* were identified across the eight iron mat samples, and all three OTU sequences are >99% identical to the Fleming sequence, meeting the species delineation threshold for 16S rRNA gene identity (>98.6% (36)) (Table S4). After correcting the classification, we find that *L. ochracea* is present in all eight samples (0.99% to 6.68%; Table 2; Table S3), consistent with microscopy and mat morphotype observations. Furthermore, these OTUs are by far the most abundant members of the *Leptothrix–Sphaerotilus* group in all eight samples.

**TABLE 2:** Percent abundance of *Leptothrix* and *Sphaerotilus* OTUs at the Savannah River Site^*a*^

OTU	Classification	ISCOA1K	ISCOB2	ISCOC1	ISCOC1bead	MN2	MN3	DE1	DE2
OTU000006	*L. ochracea*	0.016	2.22	6.17	0.012	0.004	0.009	0.012	0.015
OTU000001	*L. ochracea*	0.012	0.013	0.044	0.003	–	5.48	3.60	1.43
OTU000011	*L. ochracea*	1.82	0.013	–	4.74	0.940	0.015	0.005	0.004
OTU000215	*L. ochracea*	–	–	0.004	0.009	–	0.239	0.026	0.007
OTU000130	*L. ochracea*	0.024	–	0.037	0.106	0.008	0.011	0.019	0.009
OTU000236	*Other Leptothrix*	–	–	–	–	–	–	0.007	0.282
OTU000257	*Other Leptothrix*	–	–	–	–	–	0.002	0.151	0.015
OTU004977	*Sphaerotilus*	–	0.004	–	–	–	0.002	–	–
OTU090995	*Sphaerotilus*	–	–	–	0.003	–	–	–	–
OTU040448	*Sphaerotilus*	–	–	–	–	–	0.002	–	–
OTU113053	*Sphaerotilus*	–	–	–	–	–	0.002	–	–
OTU124633	*Sphaerotilus*	–	–	–	–	–	0.002	–	–
OTU132238	*Sphaerotilus*	–	–	–	–	–	0.002	–	–
**Total**									
	*L. ochracea*	2.10	2.37	6.68	5.17	0.989	6.41	4.17	1.61
	*Other Leptothrix*	0.139	0.115	0.015	0.022	0.000	0.082	0.349	0.472
	*Sphaerotilus*	0.000	0.004	0.000	0.003	0.000	0.011	0.000	0.000

^*a*^ Relative abundance of *Leptothrix* and *L. ochracea* OTUs above 0.1% and *Sphaerotilus* OTUs above 0.01%. Total abundance of all *L. ochracea*, other *Leptothrix*, and *Sphaerotilus* OTUs. –, not detected.

Metagenomic sequencing was performed on one iron mat sample from Spruce Point and eight iron mat samples from the SRS. We used BLAST+ to search for matches to the Fleming 16S rRNA sequence for *L. ochracea* and identified full-length 16S sequences with >99% identity to the Fleming sequence in all nine assemblies. No 16S genes were associated with metagenome-assembled genomes (MAGs) described below. However, the presence of *L. ochracea* 16S genes in the assemblies confirms that *L. ochracea* was sequenced in all nine metagenomes.

**Fig 3:**
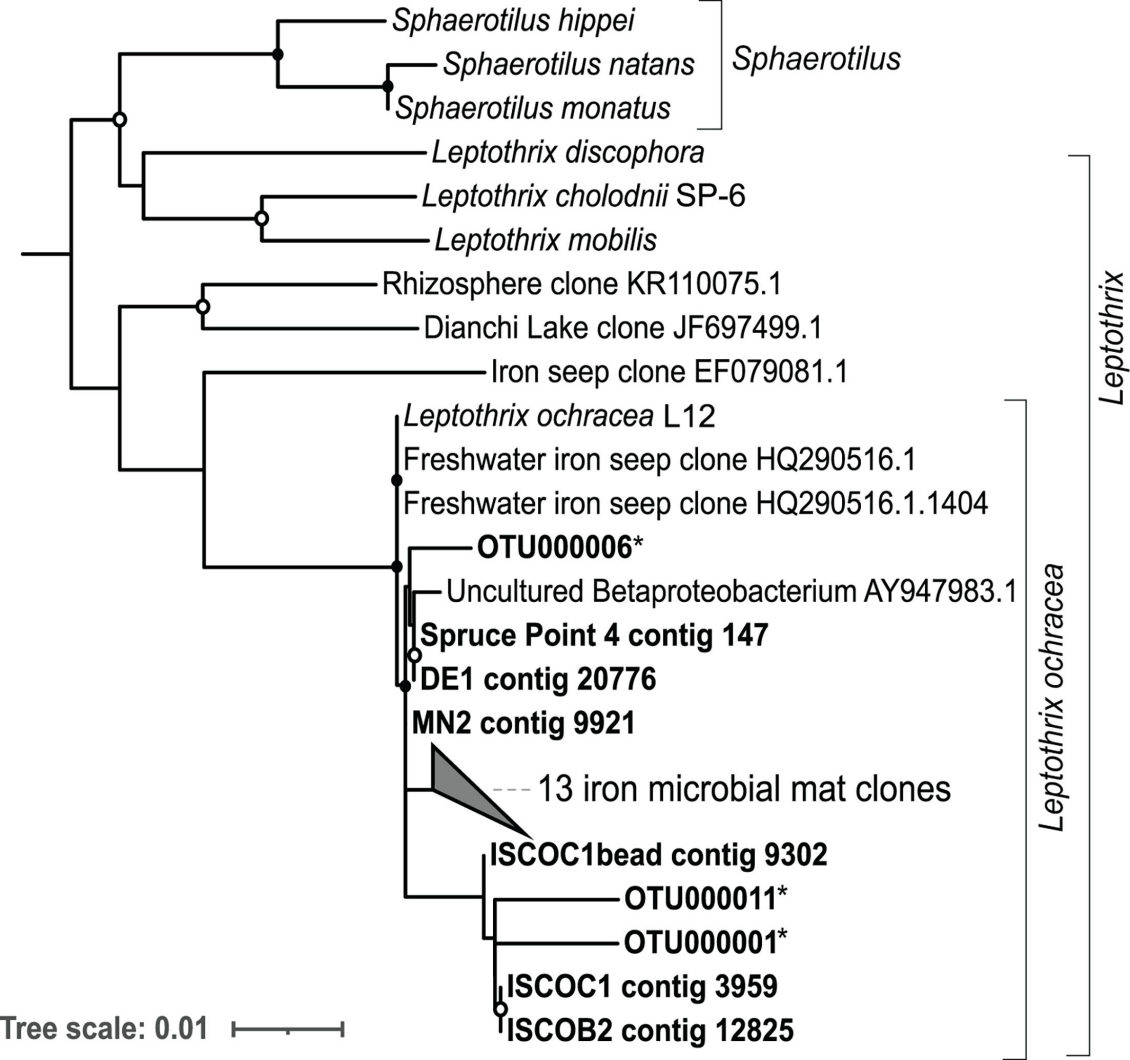
Phylogeny of 16S ribosomal RNA gene sequences in the *Leptothrix–Sphaerotilus* clade. Maximum likelihood tree of 16S rRNA gene sequences generated using RAxML with 1,000 bootstraps. Metagenome assembly and 16S amplicon sequences from this study are shown in bold. All sequences are full-length, except those denoted by asterisks, representing 465-bp 16S rRNA gene amplicon sequences. Closed circles denote bootstrap support between 75% and 100%. Open circles denote bootstrap support between 50% and 75%. *Rhodoferax ferrireducens* outgroup not shown. Accession numbers for the collapsed clade of iron microbial mat clones: LN870973.1, LN870963.1, LN870961.1, LN870953.1, LN870947.1, LN870913.1, LN870912.1, LN870911.1, LN870861.1, LN870849.1, LN870848.1, AB722229.1, AB600433.1, HQ290516.1.1404, and HQ290516.1.

A phylogenetic tree was constructed using the 16S rRNA sequences of the *Leptothrix–Sphaerotilus* group from our samples (16S rRNA amplicon sequences and metagenome sequences) and additional publicly available sequences from the *Leptothrix–Sphaerotilus* group (Fig. 3). Eighteen previously unclassified *Leptothrix* sequences were identified from SILVA and GenBank by a BLAST search against the *L. ochracea* L12 sequence. These sequences come from terrestrial freshwater iron-rich environments, including iron microbial mats and iron seeps in mines, stream and river bacterioplankton communities, and a rhizosphere (7, 37–41). Sixteen of the unclassified sequences from SILVA and GenBank were classified as *L. ochracea* based on >98.6% identity to the *L. ochracea* L12 sequence. The sequences from this study and the sequences identified by BLAST cluster with the *L. ochracea* L12 sequence, further demonstrating that they are closely related to *L. ochracea* (Fig. 3).

### *Leptothrix–Sphaerotilus* genomes

Metagenome-assembled genomes (MAGs) were reconstructed from the metagenome assemblies and manually curated using Anvio (42). One MAG from Spruce Point and twelve MAGs from the SRS were classified by GTDB-tk (43, 44) (Table 3) to the order level as Burkholderiales, which contains the *Leptothrix–Sphaerotilus* group, but none were specifically classified as *Leptothrix*. GTDB contains only two *Leptothrix* genomes (*L. cholodnii* and *L. mobilis*), which does not span the full diversity of *Leptothrix*, so GTDB may have difficulty classifying *L. ochracea*. Eleven of these Burkholderiales MAGs were deemed to be of high quality using CheckM (>90% completeness, <5% contamination) (43) and were used for further classification.

**TABLE 3:** *Leptothrix–Sphaerotilus* genome attributes

Classification	Genome name	Completeness	Contamination	Sequence size (Mb)	No. of contigs	GC content (%)	N50	L50	No. of CDS	Source
*Leptothrix ochracea*	ISCOC1.001	99.03	1.03	2.90	113	60.9	52206	15	2720	Stream
	ISCOA1K.005	97.13	2.25	2.75	291	61.1	16360	49	2678	Stream
	ISCOC1bead.006	99.03	0.47	2.91	114	60.8	64115	14	2718	Stream
	ISCOB2.007	98.18	1.87	2.59	248	61.3	19293	39	2453	Stream
	DE2.004	97.94	0.00	2.94	42	60.8	92174	8	2677	Wetland
	MN3.008	95.75	0.86	2.62	215	61.1	15561	49	2508	Wetland
	MN2.012	99.50	0.00	3.00	63	60.7	138756	7	2793	Wetland
	DE1.021	99.27	0.00	2.96	50	60.8	114507	9	2703	Wetland
	4SP.002	99.35	0.00	3.04	47	60.6	86114	11	2786	Drainage channel
	*Leptothrix ochracea* L12	23.28	0.00	0.51	36	58.0	20545	8	562	Freshwater
Other *Leptothrix*	DE1.014	100.00	0.59	4.69	65	65.1	121859	11	4441	Wetland
	DE1.018_1	34.99	8.89	2.07	276	65.2	9573	56	2230	Wetland
	DE1.018_2	61.21	34.48	5.07	792	69.6	7506	204	5173	Wetland
	*Leptothrix cholodnii* SP-6	100.00	0.00	4.91	1	68.9	N/A	1	4446	Isolate
	*Leptothrix mobilis*	100.00	0.47	4.65	15	69.0	719329	3	4141	Isolate
*Sphaerotilus*	MN3.004	90.47	1.43	3.94	418	68.0	12007	101	3954	Wetland
	*Sphaerotilus natans*	99.97	0.00	4.63	57	69.9	161150	9	4222	Isolate

**Fig 4:**
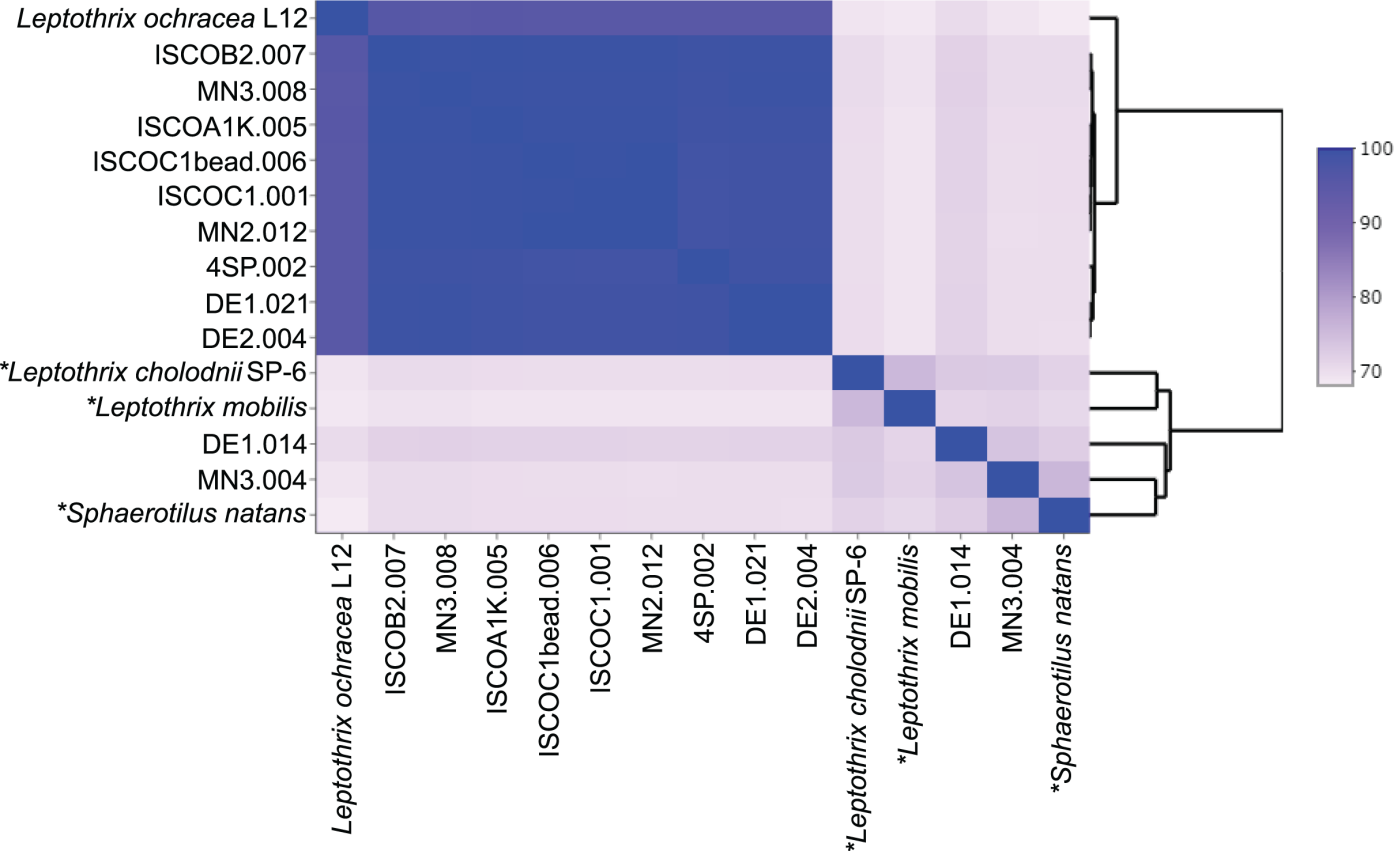
Heatmap of amino acid identity (%) between *Leptothrix* and *Sphaerotilus* genomes. Asterisks denote isolate genomes.

These MAGs are all relatively abundant in their respective assemblies (Table 4), and their relative abundance matches that of the *L. ochracea* OTUs in the 16S rRNA gene data. Given that *L. ochracea* represented the most prevalent *Leptothrix* or *Sphaerotilus* OTUs, we performed further classification to investigate whether these abundant MAGs represented *L. ochracea*. None of these MAGs contain a 16S rRNA gene, so we analyzed average amino acid identity (AAI) to determine their relatedness to *L. ochracea* L12 (Fig. 4). Nine MAGs were identified as *L. ochracea* based on the AAI values (95.3%–95.7%) above the species delineation cutoff (>95% (41)). Furthermore, the AAI values between all nine MAGs are >99%, demonstrating that these MAGs are all highly related to each other. This is consistent with findings of the study by Fleming *et al*. (7) who found very limited microdiversity in *L. ochracea* 16S rRNA gene sequences. The high relatedness of the MAGs from this study is especially striking, given their reconstruction from diverse environments at four different sampling sites from two geographically distinct locales.

**TABLE 4:** Percent abundance of *Leptothrix* and *Sphaerotilus* MAGs in their respective metagenome assemblies^*a*^

Metagenome assembly	MAG	Percent abundance
4SP	4SP.002	65.9
ISCOA1K	ISCOA1K.005	4.65
ISCOB2	ISCOB2.007	4.57
ISCOC1	ISCOC1.001	12.9
ISCOC1bead	ISCOC1bead.006	12.5
MN2	MN2.012	2.42
MN3	MN3.008	19.2
DE2	DE2.004	7.90
DE1	DE1.021	14.0
	DE1.014	4.24
	DE1.018_1	1.38
	DE1.018_2	1.26
MN3	MN3.004	2.51

^*a*^ Percent abundance values represent estimated percent community abundance including unbinned contigs.

**Fig 5:**
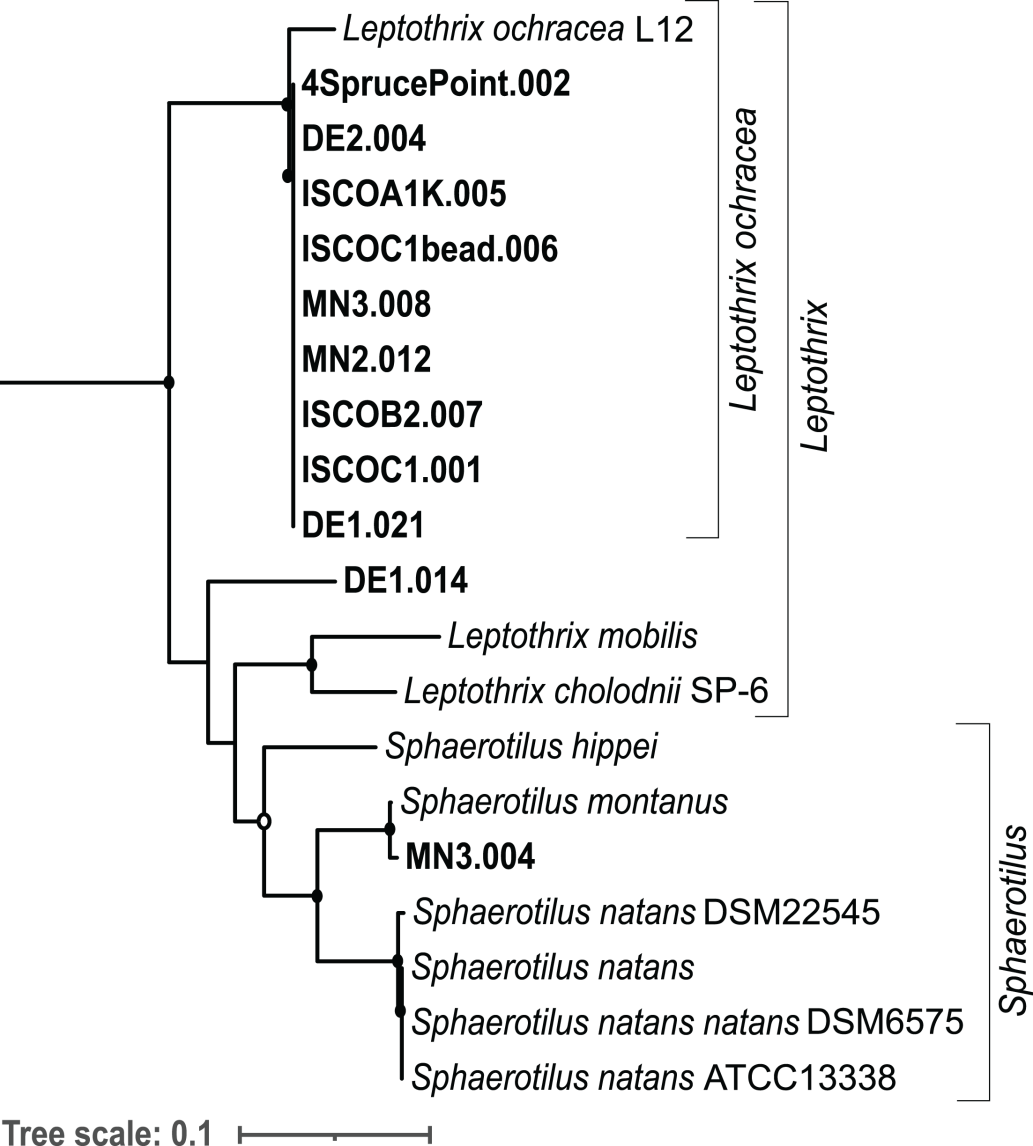
Phylogeny of concatenated ribosomal protein sequences from the *Leptothrix–Sphaerotilus* group. Maximum likelihood tree of genes from metagenome-assembled genomes and reference genomes in the *Leptothrix–Sphaerotilus* group, using *Rhodoferax ferrireducens* T118 as an outgroup. Generated using RAxML with 1,000 bootstraps from alignment of ribosomal proteins L2, L3, L4, L6, L13, L17, L19, L20, L27, L28, L35, S2, S3, S8, S9, S11, S13, and S16. Closed circles denote bootstrap support between 75% and 100%. Open circles denote bootstrap support between 50% and 75%.

We further resolved the phylogeny of these MAGs within the *Leptothrix–Sphaerotilus* group using a tree of 18 concatenated ribosomal proteins (Fig. 5). The concatenated tree confirmed the AAI results; the same nine MAGs clustered together and formed a monophyletic group with *Leptothrix ochracea* L12, while the remaining two clustered with *Sphaerotilus natans* and *Leptothrix cholodnii* SP-6 (Fig. 4 and 5). There is 100% bootstrap support for the branch separating *L. ochracea* L12 and the nine *L. ochracea* MAGs from the other members of the *Leptothrix–Sphaerotilus* group (Fig. 5). Taken together, these results confirm that *L. ochracea* is a distinct species within the *Leptothrix–Sphaerotilus* clade. The metagenome abundance, phylogenetic tree, and amino acid identity results agree with morphological and 16S rRNA gene evidence that *L. ochracea* is present and abundant in these samples and confirm that these MAGs are in fact *L. ochracea*. Thus, these nine MAGs represent the first near-complete genomes of *L. ochracea*.

The genomes of *L. ochracea* reconstructed here are similar in size (2.6–3.0 Mb) to those of the autotrophic iron oxidizers in the family Gallionellaceae. In contrast, other members of the *Leptothrix–Sphaerotilus* group have larger genomes of 4.4–5.1 Mb. The smaller genome size of *L. ochracea* is consistent with it having a distinct, more limited metabolism compared to its closest relatives.

### *L. ochracea* metabolic potential

**Fig 6:**
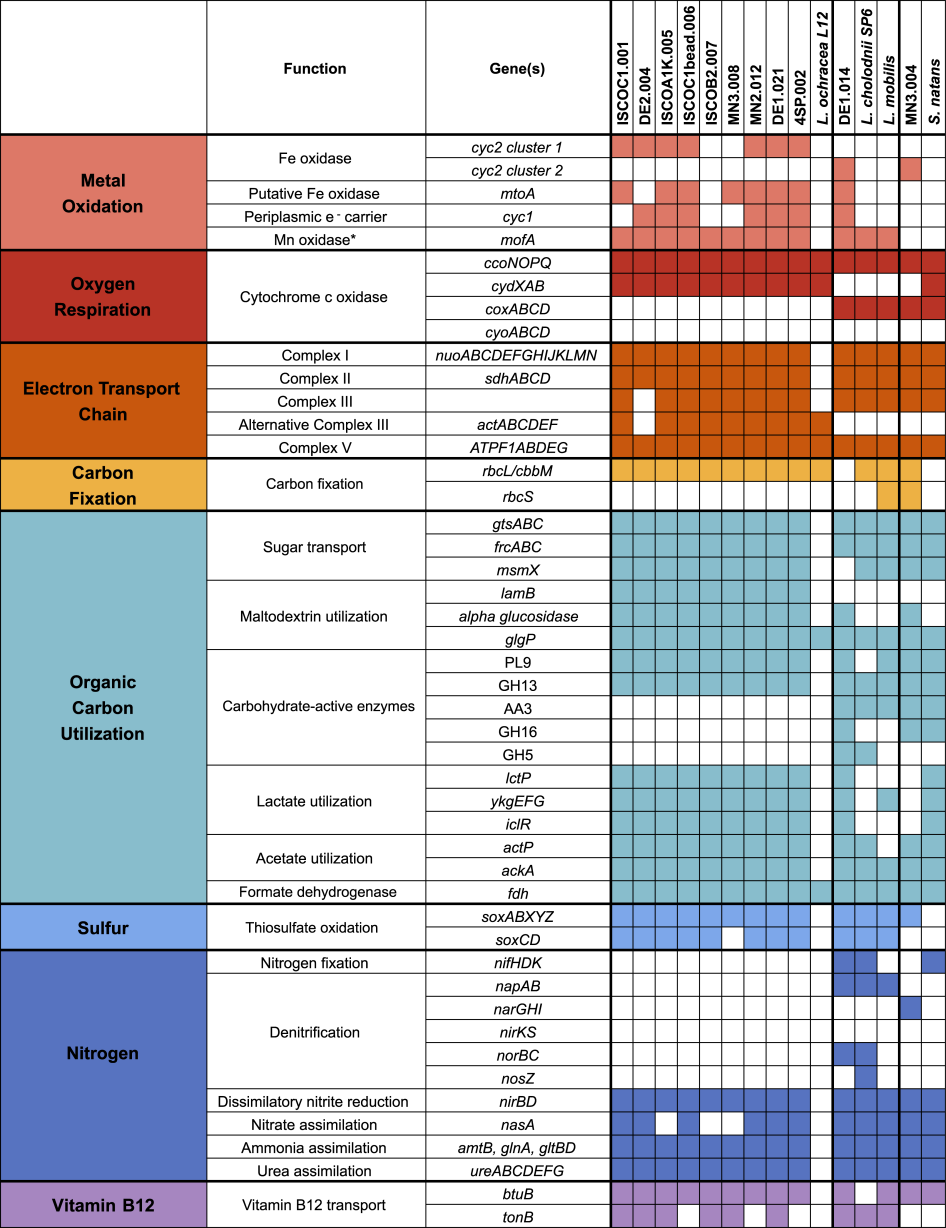
Genomic potential of the *Leptothrix–Sphaerotilus* group. Shaded boxes indicate gene presence. White boxes indicate gene absence. The asterisk indicates that *mofA* may play a role in Fe oxidation.

Genomes of *L. ochracea* were analyzed to explore their energy metabolisms and metabolic contributions to elemental cycling with a focus on genes for metal oxidation, oxygen tolerance, electron transport, and carbon assimilation. This allowed us to understand the niche of *L. ochracea* by identifying its major carbon and energy sources and comparing and contrasting these with other *Leptothrix–Sphaerotilus*. We also investigated genes for nitrogen and sulfur metabolisms to understand the breadth of biogeochemical transformations mediated by *L. ochracea*. Genes are summarized in Fig. 6 and Table S5.

#### Metal oxidation

##### Iron oxidation

Functionally, *L. ochracea* has always been categorized as a member of the iron-oxidizing bacteria (3, 45). Although it catalyzes biotic iron oxidation, it has never been shown definitively to conserve energy from iron oxidation. Furthermore, the *L. ochracea* L12 SAG lacks known iron oxidase genes (4, 46, 47). We analyzed these near-complete genomes for genes that may be responsible for iron oxidation coupled to energy conservation. Seven of the *L. ochracea* MAGs contain an iron oxidase gene, including a cluster 1 *cyc2* and/or *mtoA*, whereas the other members of the *Leptothrix–Sphaerotilus* group contain only cluster 2 *cyc2* genes (Fig. 6) (clusters defined by McAllister *et al*., (48)). The cluster 1 Cyc2 is functionally validated as an iron oxidase in neutrophilic iron oxidizers (47), while the cluster 2 Cyc2 function was demonstrated in an acidophile (49). Several of the *Leptothrix* genomes also have homologs of the gene that encodes the periplasmic electron carrier Cyc1 (Fig. 6)*,* which carries electrons from the outer membrane protein Cyc2 to the inner membrane and electron transport chain. This gene is not as prevalent among iron oxidizers as the iron oxidase genes, likely because iron-oxidizing bacteria use diverse periplasmic electron carriers. The presence of iron oxidases and periplasmic electron carrier genes gives *L. ochracea* the potential to connect iron oxidation to the electron transport chain with the same mechanism used by Gallionellaceae. This is compelling evidence that *L. ochracea* has the means to use iron oxidation for energy and growth.

The presence of these validated iron oxidase genes also makes *L. ochracea* distinct from the isolate *L. cholodnii* SP-6. Although this isolate demonstrates iron oxidation activity in laboratory cultures (46, 47), its genome lacks known iron oxidase genes, and there is no good evidence thus far for iron oxidation in *L. cholodnii* being an energy-generating process. It is unclear why *L. ochracea* and *L. cholodnii* would have different iron oxidation mechanisms, but it is likely due to the distinct roles of Fe oxidation in their metabolisms.

The presence of multiple iron oxidase genes (*cyc2* and *mtoA*) in *L. ochracea* genomes may enable the cells to utilize different ferrous iron substrates. Gallionellaceae genomes also commonly contain both *cyc2* and *mtoA* (50), and their expression differs during oxidation of dissolved and solid Fe(II) substrates (51, 52). Cyc2 is a monoheme outer membrane cytochrome–porin that most likely interacts with aqueous Fe^2+^ (47). MtoA is a decaheme cytochrome, also in the outer membrane, with a structure that could theoretically contact a solid substrate (46). In fact, *mtoA* has been shown to be expressed specifically when the Gallionellaceae *Sideroxydans lithotrophicus* ES-1 oxidizes solid Fe(II) in smectite clay (52). In *L. ochracea* iron mats, the primary iron source is aqueous Fe^2+^ coming from anoxic groundwaters, but this Fe^2+^ may become bound to organic matter (humics and extracellular polymers) and minerals (20, 53, 54). Having different iron oxidases would allow *L. ochracea* to use these varied Fe(II) forms as sources of electrons for growth.

##### Manganese oxidation

Representatives from *Leptothrix* and *Sphaerotilus* have been shown to oxidize Mn (55–58); however, it is unknown whether *L. ochracea* can also oxidize Mn. Multicopper oxidases (MCOs) can be Mn and Fe oxidases in bacteria (59–61), among other functions. We searched for MCOs in the *L. ochracea* genomes using a custom MCO detection script to identify MCOs based on a set of copper-binding amino acid motifs (62) and then clustered hits with known MCO sequences within *L. cholodnii* SP-6 for identification. Among these, *mofA* was present in all of the *L. ochracea* MAGs, with one to three copies per genome (Fig. 6; Table S5). *L. cholodnii* SP-6 possesses several distinct MCOs that have been shown to oxidize Mn, including *mnxG* and *mcoA* (59); however, the *L. ochracea* genomes only encode *mofA* (Fig. 6; Table S5). The ubiquity of *mofA* across all *L. ochracea* genomes, often in multiple copies, suggests that it is essential to growth and survival in iron-oxidizing conditions, but it is unclear whether MofA functions as an Fe or Mn oxidase in *L. ochracea*. MofA is implicated in Mn oxidation in *L. cholodnii* SP-6 (63, 64), though MCOs also possess iron oxidation activity (59–61), so its role in iron oxidation cannot be ruled out.

### Energy metabolisms

#### Oxygen

All genomes in this study possess genes for the *cbb_3_*-type cytochrome *c* oxidase (*ccoNOPQ*), and most of them also encode genes for a cytochrome *bd* ubiquinol oxidase complex (*cydABX*) (Fig. 6; Table S5). These terminal oxidases have high affinity for oxygen and therefore are widely understood to be used under microaerobic conditions (65, 66). The *L. ochracea* MAGs only encode these two terminal oxidases, whereas the other *Leptothrix* and *Sphaerotilus* genomes possess genes for the *aa_3_*-type cytochrome *c* oxidase (*coxABCD*) as well, which has low affinity for oxygen and is highly expressed under oxic conditions (67–69). This suggests that *L. ochracea* tends to occupy more microaerobic niches, while other members of the *Leptothrix–Sphaerotilus* group can grow under a wider range of oxygen concentrations.

#### Electron transport chain

All of the genomes analyzed in this study have genes for complete electron transport chains, including the *nuoABCDEFGHIJKLMN* gene cluster encoding Complex I, *sdhABCD* encoding Complex II, a complete cytochrome *bc_1_* complex (Complex III), at least one cytochrome *c* oxidase (Complex IV), and all five subunits of an F-type ATPase (Complex IV) (Fig. 6; Table S5). We confirmed the presence of genes for ACIII (*actABCDEF*) in the *L. ochracea* L12 genome, as reported by Fleming *et al*. (4) and identified its presence in eight of the nine *L. ochracea* MAGs. It is absent in all of the other *Leptothrix* and *Sphaerotilus* genomes. In all of the *L. ochracea* genomes with ACIII, *actA* was truncated. Fleming *et al*. hypothesized that this was due to the incomplete nature of the *L. ochracea* L12 assembly, but the *actA* genes in the MAGs were of the same length. The *act* gene order (*actABCDEF*) is conserved among all the *L. ochracea* genomes possessing ACIII; the only exception is genome MN3.008, where these genes were found split between two separate contigs. There is evidence that ACIII participates in reverse electron transport (RET) during autotrophic iron oxidation by Gallionellaceae (51); however, we hypothesize that *L. ochracea* does not require RET due to its utilization of organics for energy. Organic carbon oxidation generates NADH, and therefore the energetically costly process of RET would not be required to produce reducing equivalents. Thus, the *L. ochracea* ACIII, with the truncated *actA* gene, may have a distinct function from ACIII in the chemolithoautotrophic iron oxidizers.

### Carbon fixation and heterotrophy

Here, we examine the genomes of *L. ochracea* to understand its carbon metabolism, including which substrates it is able to use for biomass or energy generation.

#### Carbon fixation and storage

All of the *L. ochracea* MAGs contain genes for Form II RuBisCO (*rbcL*/*cbbM*) and genes for a full Calvin–Benson–Bassham (CBB) cycle (Fig. 6; Table S5), which suggests that they are capable of fixing CO_2_ into organic carbon. This demonstrates that, despite the general understanding of members of the *Leptothrix–Sphaerotilus* group as heterotrophs, *L. ochracea* has the potential to use inorganic carbon sources. *L. ochracea* was shown to assimilate CO_2_ at a small fraction of its total carbon demand (4). This implies that, while it may obtain some carbon by CO_2_ fixation, it can also assimilate organic carbon. Combined, this genomic and experimental evidence points to a mixotrophic metabolism.

The genes for RuBisCO and the CBB cycle are not unique to *L. ochracea*, as the other members of the *Leptothrix–Sphaerotilus* group also possess the majority of the pathway (Fig. 6; Table S5). This reveals that they also have the potential to use inorganic carbon sources; however, they thrive in environments with higher quantities of organics than *L. ochracea* (32). Thus, they could alternatively utilize the CBB cycle to recycle CO_2_ produced during heterotrophic metabolism (70–72).

#### Organic carbon utilization

##### Simple sugars and polysaccharides

All complete genomes in this study possess genes for glycolysis and gluconeogenesis and a number of simple sugar transporters (Fig. 6; Table S5). These include genes for a glucose/mannose transport system (*gtsABC*), a fructose transport system (*frcABC*), and at least one gene for a putative multiple sugar or simple sugar transport system. Additionally, *L. ochracea* contains genes for the import and degradation of maltohexose (maltodextrin), including a maltoporin for import, and alpha glucosidase and glycogen phosphorylase for degradation of maltodextrin into D-glucose monomers. The ability to import this polysaccharide would afford *L. ochracea* the ability to use carbon present from decomposing organic matter in wetlands; therefore, these genes suggest that *L. ochracea* is able to capitalize on simple sugars and polysaccharides from organic matter degradation and use them for energy and/or biosynthesis pathways.

In addition to genes for sugar import, the *L. ochracea* genomes encode for numerous genes with CAZy (carbohydrate active enzymes) annotations including polysaccharide lyases (PL9) and glycoside hydrolases (GH13) (Fig. 6; Table S5). Despite these annotations, BLAST searches did not reveal close homologs to these sequences with verified functions. Representatives from category PL9 can degrade major plant cell wall polysaccharides (73) and the sheaths of *Sphaerotilus natans* (74). Genomes from the other *Leptothrix* and *Sphaerotilus* representatives possess higher copy numbers of the PL9 and GH13 CAZy genes, in addition to numerous genes in the category auxiliary activities (AA3), and glycoside hydrolases (GH16 and GH5) (Table S5). Overall, *L. ochracea* has more limited organic carbon utilization pathways than other *Leptothrix–Sphaerotilus*, consistent with differences in growth responses to organics. Isolates of the other *Leptothrix–Sphaerotilus* have strong growth responses to organic carbon and also produce more robust organic sheaths composed of polysaccharides, in contrast to *L. ochracea*, in which sheaths are primarily iron oxyhydroxide (9, 13, 19, 35, 75–77). *L. ochracea* sheath synthesis may demand less organic input and therefore fewer organic utilization pathways.

##### Organic acids and other substrates

All *L. ochracea* MAGs have genes for L-lactate permease (*lctP*) and L-lactate dehydrogenase (*ykgEFG*), which transform L-lactate into pyruvate (Fig. 6; Table S5). This indicates that *L. ochracea* is able to import lactate and feed it directly into central metabolism. All of these genomes also have a predicted lactate-responsive regulator gene (*iclR*), which may indicate that they are able to detect the presence of lactate and express these genes when it is present, and use lactate for energy and/or biosynthesis. Genes for acetate permease (*actP*) and acetate kinase (*ackA*) are also present in all nine *L. ochracea* MAGs (Fig. 6; Table S5), suggesting that acetate can be imported and shuttled into pyruvate metabolism. These genes suggest that *L. ochracea* has the potential to use both lactate and acetate for energy generation and to assimilate organic carbon from them via TCA cycle intermediates.

All *Leptothrix* and *Sphaerotilus* genomes contain numerous transporters for peptides and amino acids and peptidases (Table S5), suggesting that they are capable of utilizing peptides and amino acids as sources of carbon. All genomes in the *Leptothrix–Sphaerotilus* group contain genes for NAD-dependent formate dehydrogenase (Fig. 6; Table S5). Formate is often present in wetlands as a product of fermentation, and formate dehydrogenase can regenerate NADH using formate (78). This suggests that formate can be an energy-generating substrate for *L. ochracea* and other *Leptothrix* and *Sphaerotilus* members.

### Other metabolisms

#### Sulfur oxidation

All of the complete *Leptothrix* and *Sphaerotilus* genomes possess genes for thiosulfate oxidation to sulfur (*soxABXYZ*), except *Sphaerotilus natans*, and most also have *soxCD* for oxidation to sulfate (79) (Fig. 6; Table S5). This is consistent with the ability of other iron oxidizers to oxidize thiosulfate (e.g., *Sideroxydans lithotrophicus* ES-1 and *Sideroxyarcus emersonii* MIZ01) (51, 80). These genes give *L. ochracea* the ability to use sulfur species as electron donors for energy conservation, in addition to iron and organics. Furthermore, these genes suggest that *L. ochracea* contributes to biogeochemical sulfur cycling via thiosulfate oxidation.

#### Nitrogen metabolism

None of the *L. ochracea* MAGs contain genes for nitrogen fixation (*nifHDK*) (Fig. 6; Table S5), indicating that *L. ochracea* is not capable of nitrogen fixation. Genes for denitrification (*napA*, *narGHI*, *nirK*, *nirS*, *norBC,* and *nosZ*) are largely absent from *Leptothrix ochracea* (Fig. 6; Table S5); however, all complete genomes in this study contain genes for a respiratory nitrite reductase (*nirBD*), suggesting that the *Leptothrix–Sphaerotilus* group may conduct dissimilatory nitrite reduction during aerobic growth (81). All *Leptothrix* and *Sphaerotilus* genomes also possess several genes for nitrogen assimilation, including *nasA* for assimilatory nitrate reduction, genes for ammonia import and assimilation (ammonia transporter, glutamine synthetase, and glutamate synthase), and genes encoding urease (*ureABCDEFG*) for urea assimilation (Fig. 6; Table S5). In summary, these results demonstrate that the main contribution of *L. ochracea* to nitrogen cycling is through dissimilatory nitrite reduction and through assimilation of nitrate, ammonia, and urea.

#### Hydrogen oxidation

Genes for [NiFe]-hydrogenases (*hypABCDEF*, *hupUV*, *hoxFU*, *hoxHY*) are absent in all *Leptothrix ochracea* genomes, but are encoded in the genomes of other members of *Leptothrix* and *Sphaerotilus* (Table S5).

#### Vitamin B12

It has been widely reported that vitamin B12 is a growth requirement for the *Leptothrix–Sphaerotilus* group and must be supplemented in growth media (5, 27, 55, 82). Thus, we looked for vitamin B12 import pathways in these genomes. A vitamin B12 transporter gene (*btuB*) was identified in all of the *Leptothrix* and *Sphaerotilus* genomes, except for *L. ochracea* L12 and *L. cholodnii* SP-6 (Fig. 6; Table S5). This transporter performs active transport of vitamin B12 via coupling with the protein TonB (83–85). The *tonB* gene was present in the majority, though not all, of the *Leptothrix* and *Sphaerotilus* genomes that contained *btuB. L. cholodnii* SP-6 lacks the *btuB* transporter and is the only *Leptothrix* or *Sphaerotilus* genome that possesses genes involved in biosynthesis of vitamin B12 (Fig. 6; Table S5). These results support the suggestion that vitamin B12 is a requirement for most members of this group, but that it cannot be considered a defining trait of the *Leptothrix–Sphaerotilus* group.

### Transcriptome

Metatranscriptome sequencing was performed on SRS mat samples DE1, MN3, and ISCOA1K. We mapped transcript reads to the *L. ochracea* MAGs from these three samples (DE1.021, MN3.008, and ISCOA1K.005) to gain an insight into the metabolic activity of *L. ochracea* in its typical environments. The transcriptomes are consistent with our interpretation of *L. ochracea* as a metal-oxidizing, sheath-forming organism that can tolerate oxygen and oxidative stress (e.g., *mofA, epsABP*, and glutathione peroxidase gene, Table 5). The expression of genes for iron oxidation, as well as carbon fixation and utilization, supports our characterization of *L. ochracea* as a mixotrophic iron oxidizer. MAGs DE1.021 and ISCOA1K.005 highly expressed iron oxidation genes *cyc2* and *cyc1*, while all three MAGs show the expression of the multiheme iron oxidase gene *mtoA* and various other cytochromes (Table 5; Table S6 to S8). The MCO *mofA* was one of the top expressed genes in all three MAGs (Table 5). Although the role of *mofA* in *L. ochracea* is unclear, we can gain insight by examining the environmental chemistry. Iron concentrations (measured by ICP-MS) were much higher than Mn in surface water at sites DE1 and MN3 and in sediment porewater at site MN3. Mn concentrations were 0.7–1.2 µM in surface water and 1.4–2.0 µM in porewater, whereas Fe concentrations were 4–11 µM in surface water and 13–27 mM in sediment (Table S1). The abundance of Fe relative to Mn implies the utility of *mofA* in iron-rich environments and may suggest its role in Fe oxidation. Furthermore, the *cbb_3_*-type cytochrome c oxidase genes *ccoNO* are expressed highly in all three MAGs (Table 5; Table S6 to S8). A number of organic carbon utilization genes are expressed by *L. ochracea*, including high expression of genes for lactate permease, lactate dehydrogenase, and a sodium/acetate symporter, and moderate expression of the glucose/mannose transport system *gtsABC* (Table 5; Table S6 to S8). On the other hand, *rbcL* (RuBisCO) expression is low to moderate (Table 5; Table S6 to S8). These function-based results provide strong evidence that *L. ochracea* is growing as an aerobic, mixotrophic iron oxidizer in the environment.

**TABLE 5:** *In situ* expression of key genes in *L. ochracea* MAGs from the three SRS sampling sites

Function	Gene	Percentile expression^*a*^		
		DE1.021	ISCOA1K.005	MN3.008
Iron oxidation	*cyc2*	99.6	99.3	N/A^*c*^
Iron oxidation	*cyc1*	90.7	94.4	N/A^*c*^
Iron oxidation	*mtoA*	82.3	60.9	31.3
cbb3-type cytochrome c oxidase	*ccoN*	97.2	98.8	96.5
cbb3-type cytochrome c oxidase	*ccoO*	96.7	99.1	96.8
Glutathione peroxidase/oxidative stress response	*btuE*	91.8	92.0	84.8
Lactate permease	*lctP*	89.9	88.6	98.0
Lactate dehydrogenase	*ykgEFG*	82.0	74.2	64.5
Na+/acetate symport	*actP*	81.3	89.1	88.8
Glucose/mannose transport	*gtsA*	74.0	73.8	68.4
Glucose/mannose transport	*gtsB*	60.1	57.5	54.4
Glucose/mannose transport	*gtsC*	35.4	30.9	21.4
RuBisCO	*rbcL*	28.2	50.1	56.1
Polysaccharide export protein wza^*b*^	*epsA*	93.5	93.6	92.4
Polysaccharide export protein wzb^*b*^	*epsP*	53.5	46.5	41.8
Polysaccharide export protein wzc^*b*^	*epsB*	69.7	81.4	70.3
Metal oxidation	*mofA* copy 1	97.2	99.4	97.7
Metal oxidation	*mofA* copy 2	76.4	90.7	3.56

^*a*^ Percentile expression represents the rank of each gene’s mapped read count in comparison to all expressed genes (read counts > 0). Values represent the percentage of genes with read counts lower than that gene.

^*b*^ Putative involvement in sheath formation.

^*c*^ N/A indicates gene absence.

### *Leptothrix ochracea* metabolic model

**Fig 7:**
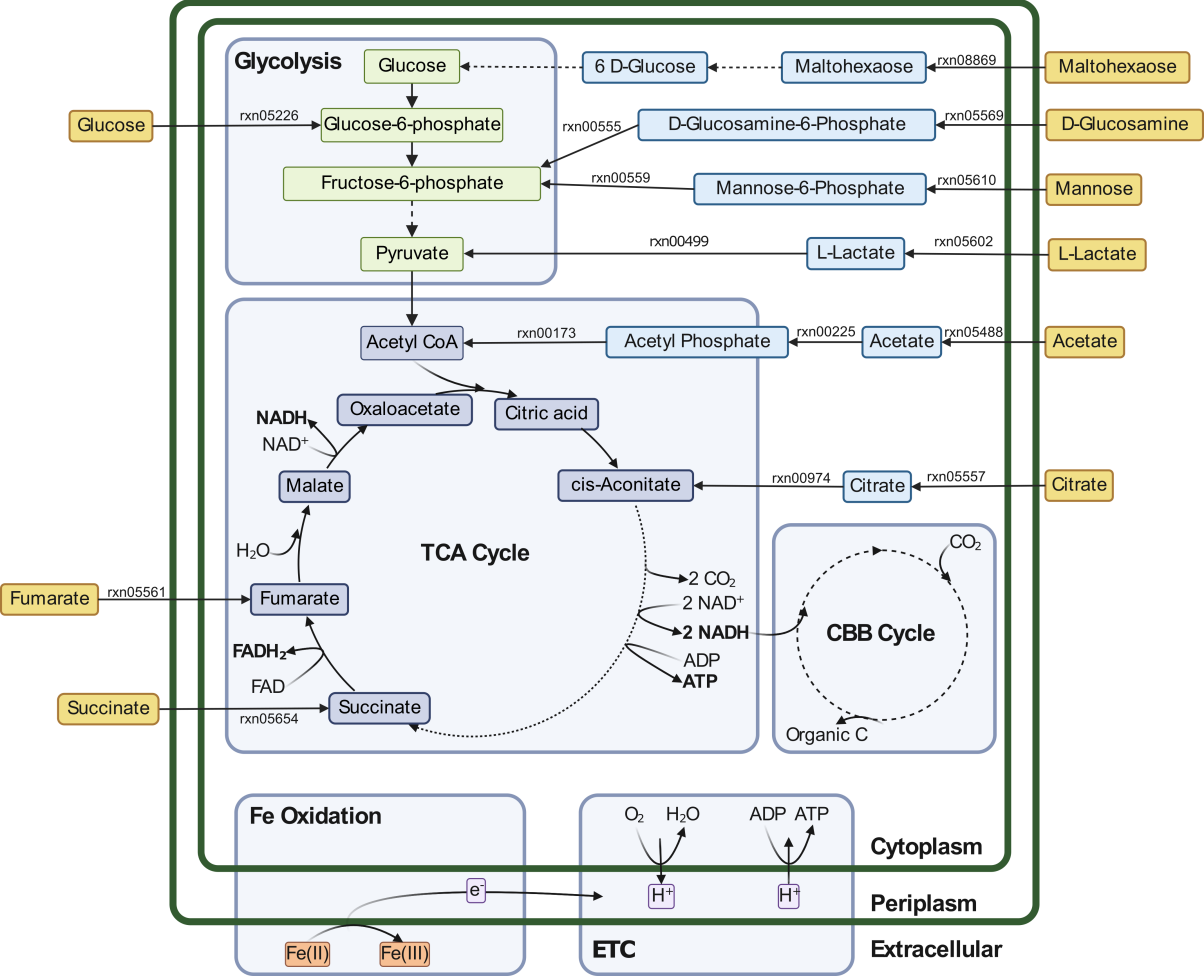
*In silico* metabolic model of *Leptothrix ochracea* MAG DE1.021 on complete media. Reactions pictured illustrate selected import and central metabolic pathways to represent a subset of reactions captured in the full model. Reaction numbers (rxn#) correspond to unique reaction identifiers in the ModelSEED biochemistry database. Created with BioRender.com.

In order to gain an additional insight into the activity and metabolism of *L. ochracea*, *in silico* stoichiometric metabolic models of *L. ochracea* MAG DE1.021 were computed using the ModelSEED tools in KBase. To narrow down which organic carbon compounds *L. ochracea* is capable of importing and utilizing for growth, we constructed the first model using complete media (i.e., media containing all available compounds in the ModelSEED database) and manually added the energy-generating iron oxidation pathway to the model. When *L. ochracea* DE1.021 was given complete media, the model displayed flux through the import of numerous organic carbon substrates: lactate, fumarate, glucose, succinate, glucosamine, mannose, maltohexaose, citrate, and all 20 amino acids. These compounds are fed into central metabolism and used for energy and biosynthesis (Fig. 7). Importantly, genes for these import reactions and downstream pathways are indeed present in the *L. ochracea* genome and not an artifact of gapfilling the model. Furthermore, adding iron oxidation to the complete media model resulted in an increase in theoretical biomass yield (Table 6; objective value).

**TABLE 6:** Objective values and exchange flux for *L. ochracea* metabolic models

C_org_ source	Objective value (without Fe oxidation)	Objective value^*a*^ (with Fe oxidation)	C_org_ substrate exchange flux^*b*^
Complete media	72.5	82.5	N/A^*c*^
Glycolate	0.603	0.577	1
Maltohexaose	1.47	1.41	1
Formate	0.603	0.577	1
D-Glucose	0.747	0.715	1
Succinate	0.675	0.658	1
Fumarate	0.663	0.646	1
Acetate	0.603	0.577	1
D-Mannose	0.603	0.715	1
L-Lactate	0.603	0.623	1

^*a*^ Objective value is a proxy for biomass yield based on the calculated flux through the Gram negative biomass reaction (bio1).

^*b*^ Exchange flux for Corg substrates is set to a maximum value of 1 based on media composition.

^*c*^ N/A indicates that there are variable exchange fluxes for the multiple organic carbon substrates within the model.

To evaluate the growth yield of *L. ochracea* on diverse organic substrates individually, metabolic models of *L. ochracea* were constructed using minimal media with Fe^2+^ and single carbon sources, which were chosen based on its genomic content and the model on complete media. Models on glucose, mannose, formate, succinate, fumarate, maltohexaose, glycolate, acetate, and lactate each demonstrated biomass production, which is consistent with the presence of multiple transporters for organic acids, sugars, and dicarboxylic acids. Furthermore, iron oxidation increased the theoretical biomass yield for the models grown on mannose and lactate, suggesting that iron oxidation supplements energy production in these models (Table 6). All of these compounds were transformed into intermediates of either glycolysis or the tricarboxylic acid (TCA) cycle (Fig. 7); the flux through these central metabolic reactions in these models indicates that these compounds are being used for both energy generation and biosynthesis (Tables S11 to S14; https://figshare.com/articles/dataset/_i_Leptothrix_ochracea_i_metabolic_models_flux_balance_analysis_tables/25977355).

These results demonstrate that it is theoretically possible for *L. ochracea* to produce biomass while growing mixotrophically using these carbon sources and iron oxidation.

### Implications and conclusions

Resolving the identity and niche of *L. ochracea* requires an understanding of its carbon and energy sources. In the absence of a *L. ochracea* isolate, we used a metagenomic approach on samples from three of its typical environments at two sites. These yielded the first near-complete genomes of *L. ochracea* from which we could interpret potential metabolic capabilities and connect these to our understanding of its niche. We also used metatranscriptomics to gain an insight into the activity of *L. ochracea* in its typical environments.

Historically, *L. ochracea* has primarily been classified by the presence of sheaths in iron-rich environments (i.e., by morphological observations), but the sheaths are not live material, rather an indication of previous presence. Molecular methods are required for more precise tracking and quantification, though reliable identification requires sufficient reference sequences. Fleming *et al*. (7) identified a 16S rRNA gene sequence from *L. ochracea* using FISH and reconstructed a partial single-cell amplified genome of *L. ochracea*, providing a molecular basis with which to identify *L. ochracea*. Here, we have recovered 16S sequences from both 16S rRNA amplicon sequencing and metagenome assemblies that are closely related to the Fleming 16S sequence (>99% identity). Furthermore, we recovered nine near-complete genomes that are closely related to the *L. ochracea* L12 partial single-cell amplified genome, clustering together in concatenated ribosomal protein trees. Overall, our work expands the known 16S diversity of *L. ochracea*, as well as providing high-quality genomes, resulting in a robust reference molecular data set from multiple sites and environments, with which further *L. ochracea* can be identified. Altogether, our phylogenetic analyses demonstrate that *L. ochracea* forms a distinct group within the *Leptothrix–Sphaerotilus* clade. It is notable how closely related both the *L. ochracea* MAGs and 16S rRNA gene sequences are, despite their reconstruction from diverse sites. This suggests there may be selective pressures that resist accumulation of genetic variability.

*L. ochracea* has been shown to oxidize iron biotically, but the role of iron oxidation in its metabolism has not been established. Although *Leptothrix* and *Sphaerotilus* isolates are known as metal oxidizers, it has been debated whether Fe or Mn oxidation can be used for energy and growth (26–29). The full genomes of *L. ochracea* presented here provide the first evidence that any member of the *Leptothrix–Sphaerotilus* group has at least two characterized iron oxidase genes, *cyc2* and *mtoA*, along with other genes required to form an energy-conserving electron transport chain based on iron oxidation. These same genes are used by known autotrophic iron oxidizers to conserve energy from iron oxidation (47, 49, 51, 52). This strongly suggests that *L. ochracea* can conserve energy from iron oxidation and use this energy for growth, which could make *L. ochracea* distinct from other *Leptothrix* and *Sphaerotilus* in this ability.

*L. ochracea* appears to combine the ability to oxidize Fe for energy with the capabilities of carbon fixation and heterotrophy, making it an iron-oxidizing mixotroph. The carbon metabolism of *L. ochracea* has long been a source of debate and confusion, and thus an important outcome of this work is the multiple lines of evidence for mixotrophy based on its genome content, environmental transcriptomes, and metabolic modeling. The mixotrophic Fe-oxidizing metabolism is consistent with the work of Fleming *et al*. that showed an Fe(II) requirement for growth and incorporation of both acetate and CO_2_ into cell biomass (4). While it is unclear if *L. ochracea* is an obligate or facultative mixotroph, we suggest that its niche is tied to mixotrophy, both for carbon (autotrophy/heterotrophy) and for energy (lithotrophy/organotrophy). This would help explain seemingly disparate observations that it behaves like an autotrophic Fe-oxidizer but has a propensity for organic carbon (27, 32). The ability to use organics may enable its prodigious sheath production and therefore its role as an ecosystem engineer. Further study could elucidate the balance of energy and carbon inputs that allow *L. ochracea* to thrive in terrestrial wetland environments alongside autotrophs and heterotrophs, taking advantage of organic breakdown products and creating massive iron mats that host communities and sequesters various nutrients, carbon, and metals. An important example is *Leptothrix* in Arctic tundra wetlands (86), which can play a significant role in controlling nutrient P dynamics in these critical ecosystems (87) via binding P to its sheaths (14). Ultimately, elucidating the metabolic connections between *L. ochracea* and iron mat communities will improve our understanding and predictions of wetland biogeochemical cycles.

## MATERIALS AND METHODS

### Sampling sites

Iron microbial mats were sampled from Spruce Point (Boothbay Harbor, ME, USA) in June of 2016 and from wetland and stream sites at the Savannah River Site (SRS) (Jackson, SC, USA) in May of 2021 and January of 2022. Within the Savannah River watershed, Tims Branch is a second-order stream surrounded by forested riparian wetlands. In the stream, prolific iron mats are found in low-flow areas along the edges of the stream where iron-rich groundwater discharges into surface water. Iron mats in Tims Branch grow in patches of between a few cm to ~1 m in size. In the SRS wetlands, mat growth occurs in low-flow areas and standing water. In some sections of these wetlands, mat growth extended continuously across areas of up to 60 square meters. The second site, the Spruce Point site, is a narrow rock-lined 25-meter-long roadside drainage channel adjacent to a wetland. The water is shallow (10–20 cm), and the entire trench is often filled with iron mats dominated by the sheaths of *L. ochracea*.

Samples were taken from the following three sites at the SRS and from one site at Spruce Point:

1. MN site (33.3375°N 81.7186°W): The MN site at the SRS is a wetland adjacent to Tims Branch with pockets of iron mat growth, especially in low-flow areas downgradient from trees.
2. DE site (33.3392°N 81.7181°W): The DE site at the SRS is a wetland adjacent to Tims Branch, upstream from the MN site, which displays extensive iron mat growth 5–8 cm thick.
3. ISCO site (33.3164°N 81.7138°W): The ISCO site is an SRS stream site within Tims Branch that displays several areas of *Leptothrix*-type mats.
4. SP site (43.8294°N 69.6256°W): Spruce Point is a shallow freshwater drainage channel with slow water flow, which displays fluffy mat growth in puffballs and small channels.

### Geochemical analyses

#### Savannah River site

A Hanna HI9829 multiparameter meter was used to record GPS coordinates and measure pH, oxidation reduction potential, percent dissolved oxygen, electrical conductivity, and temperature in the surface water. Oxygen was also measured in the surface water and below the surface of iron mats using a HI9142 dissolved oxygen meter.

Surface water samples were filtered in the field using 0.2-µm nylon filters. Filtered water samples for Fe(II) measurements were acidified in 40 mM sulfamic acid in the field to limit Fe oxidation. The following chemical analyses were conducted the same day at the on-site laboratory: (1) nitrite measurements using a Hach Spectrophotometer with the NitriVer 3 Nitrite Reagent at 507 nm (2); dissolved Fe^2+^ measurements using a ferrozine assay (88) read at 562 nm on a Hach Spectrophotometer. Additional surface water samples were filtered in the field using 0.45-µm nylon filters. Filtered samples for analysis of anions and metals were preserved at 4°C and later analyzed at the University of Delaware Advanced Materials Characterization Laboratory. Anion concentrations were measured by ion chromatography using the Metrohm IC Pro. Filtered samples were acidified in 2% nitric acid, and metal concentrations were measured via ICP-MS.

A semipermanent passive diffusion sampler was placed in the wetlands surrounding Tims Branch at the MN3 site in April 2021, as described by MacDonald *et al*. (89) and Kaplan *et al*. (90). The samplers consisted of 60-mL chambers stacked vertically with a 0.22-µm pore size polyethersulfone membrane separating the chambers from the sediment. Each sampling chamber was connected aboveground by two 0.16-cm Tygon tubes, which were used to inject deoxygenated, deionized water into each chamber. Sediment porewater and chamber water were allowed to equilibrate for 6 months before withdrawing water samples using the Tygon tubes. One advantage of this sampler is that multiple samples can be readily collected at the same location without disturbing the sediment. Diffusion samplers placed at 46, 43, 41, and 35 cm below the sediment surface were sampled in this study. Samples from each chamber were preserved for ion chromatography, ICP-MS, and dissolved Fe^2+^ measurements as described above.

#### Spruce Point site

The surface water pH was 6.05, and the water temperature was 15°C. Dissolved oxygen was measured by using a Pyroscience Firesting optical oxygen probe. Dissolved Fe^2+^ measurements were performed using a ferrozine assay (88). Water was filtered through a 0.22-µm syringe filter and diluted 1:10 directly into ferrozine.

### Biological sample collection and preservation

Iron mats were sampled from wetlands and streams using sterile serological pipets and dispensed into sterile 50-mL conical tubes. Mat material was allowed to settle for several minutes. The supernatant was then decanted, and the remaining mat material was homogenized. One-mL aliquots of the homogenized mat were placed into 2-mL tubes and flash-frozen on dry ice in the field for DNA extraction. One 17-L bulk mat sample (DE2) was collected into a carboy using a Global Water SP200 Variable Speed, Peristaltic, Fluid Sampling Pump. The bulk mat was allowed to settle overnight before further sampling. The settled mat material was homogenized, sampled, and flash-frozen on dry ice. At the SP site, approximately 15 mL of the light-colored mat material was carefully collected using a sterile 1-mL pipette tip with the end cut-off to enlarge the opening. This was done to ensure that the most active *L. ochracea* cells at the leading edge of the mat were captured (34).

### Microscopy

Iron mats were visualized using light microscopy at the on-site laboratory to check that mats contained abundant sheath material, indicative of *L. ochracea*. After confirmation, mat samples were preserved in 4% paraformaldehyde for further microscopic analysis. Upon return to the laboratory, aliquots of these samples were treated with SYTO-13 green fluorescent nucleic acid stain and were visualized under phase contrast and fluorescence microscopy to confirm the presence of sheaths and chains of cells within the sheaths. Additional aliquots of the samples were fixed in 4% glutaraldehyde for scanning electron microscopy. Glutaraldehyde-fixed samples were washed three times and then dehydrated in an ethanol dilution series of increasing concentrations (25%, 50%, 75%, 95%, and 100%). Following dehydration, the samples were placed into a Tousimis Autosamdri-815B critical point dryer. Dried samples were mounted on aluminum stubs and coated with platinum on a Leica EM ACE600. Imaging was performed on a Thermo Scientific Apreo Volumescope SEM at 2kV.

### DNA extraction

#### Savannah River site

DNA was extracted from flash-frozen field samples using the Qiagen DNeasy Powersoil Pro kit according to the manufacturer’s instructions. To ensure that flash freezing did not impact the recovery of *L. ochracea*, two additional extractions (MN3 and DE1) were performed on fresh (never frozen) iron mat at the field site within hours of sampling. These were compared to the frozen samples based on the 16S rRNA gene sequencing data in order to select samples for metagenome sequencing. For samples DE1 and MN3, both extractions were used for 16S rRNA amplicon sequencing. The communities in frozen and fresh samples were comparable, so the fresh, unfrozen extractions were used for metagenome sequencing.

#### Spruce Point site

DNA was extracted using the Mo Bio Powersoil DNA extraction kit according to the manufacturer’s instructions, with 200 ul 25:24:1 phenol:chloroform:isoamyl alcohol solution (Sigma Aldrich) added to each bead tube, as described in Scott *et al*. (91).

### 16S rRNA amplicon and metagenome sequencing

#### Savannah River site

All 16S rRNA gene amplicon sequencing and metagenome sequencing were performed at the University of Delaware Sequencing and Genotyping Center. 16S rRNA gene libraries were prepared using the Illumina 16S Sample Preparation Guide. Amplicons were prepared using the lllumina 16S b341F and lllumina 16S 805R primers, with all preparations done at half the volume (92). Libraries for ISCO and MN2 samples were prepared with 2 ng input DNA and 40 amplicon PCR cycles. Libraries for MN3 and DE samples were prepared with 12.5 ng input DNA and 30 PCR cycles. Libraries were sequenced on an Illumina MiSeq using a Nano 500 cycle sequencing kit.

Mothur (v1.48.0) was used to process the raw 16S rRNA data (93, 94). Data were quality-controlled by filtering out low-quality sequences, removing chimeras, and removing any non-bacterial sequences classified as chloroplasts, mitochondria, archaea, or eukaryotes. OTUs were generated using a reference free approach via clustering the sequences at a 99% identity threshold. Sequences were classified taxonomically using the SILVA database (v. 138.1) (95–98). The 16S rRNA data were analyzed for the abundance of known iron-oxidizing bacteria, including *Leptothrix* OTUs. Results of the 16S rRNA analysis were then used to select samples for metagenomic sequencing.

For the metagenomic sequencing, libraries were prepared using the Illumina DNA prep library preparation kit according to the manufacturer’s instructions. Libraries for ISCO and MN2 samples were prepared with reactions done at one-third the volume (92), 11.5-minute tagmentation reaction, and eight PCR cycles. Sequencing was done via Illumina NextSeq 2000 using a 300 cycle P3 sequencing kit. Libraries for MN3 and DE samples were prepared with reactions done at one-third the volume (92), 11-minute tagmentation reaction, and 10 PCR cycles (99). Sequencing was done via Illumina NextSeq 550 using a 300 cycle high output sequencing kit. Both rounds of metagenome sequencing obtained 151-bp paired end reads.

#### Spruce Point site

Metagenomic sequencing was done via Illumina NextSeq 550 at the Integrated Microbiome Resource sequencing center. One nanogram of each fluorescently quantified sample was subjected to NexteraXT (Illumina) library preparation, as per the manufacturer’s instructions, except clean-up and normalization were completed using the Just-a-Plate 96 PCR Purification and Normalization Kit (Charm Biotech). Equal amounts of all barcoded samples (Nextera XT CDIs) were then pooled and sequenced in a shared 150 + 150 bp PE NextSeq550 run using a High-Output v2 kit. Sequencing obtained 151-bp paired-end reads.

### Metagenome assembly, binning, and annotation

Raw reads were trimmed then filtered to a minimum length of 75 bp and minimum quality of 28 using Cutadapt (100). Quality of the trimmed and filtered reads was checked using FastQC v0.11.9 (101). Paired-end reads were merged using FLASh v1.2.11 (102) with a maximum overlap read length of 151 bp. Metagenome assemblies were generated using metaSPAdes v3.15.3 with kmer lengths of 21, 33, 55, 77, 99, 111, and 127 bases (103). Binning was performed in KBase using MaxBin2.0 v2.2.4 (104), CONCOCT v1.1 (105), and MetaBAT2 v1.7 (106) with minimum contig lengths of 1,000, 2,500, and 2,500 bp, respectively. Resulting bins were optimized, and the best non-redundant bins from each program were selected, using DASTool v1.1.2 (107). CheckM v1.0.18 (108) was used to evaluate bin quality. Bin taxonomy was classified using GTDB-tk v1.7.0 (44, 109, 110).

Unclassified Burkholderiales bins were further placed into a concatenated ribosomal protein tree with known *Leptothrix–Sphaerotilus* representatives to determine which, if any, belonged to the *Leptothrix* genus. The concatenated alignments of 18 large and small ribosomal protein genes (L2, L3, L4, L6, L13, L17, L19, L20, L27, L28, L35, S2, S3, S8, S9, S11, S13, and S16) were generated, trimmed, and masked (sites > 70% gaps) in Geneious v10.2.6. These proteins were selected based on the ribosomal proteins present in the *L. ochracea* L12 genome and those used by Olm *et al*. (111) for species-level delineation. The final maximum-likelihood tree was generated using RaxML-NG v1.2.0 with the LG + G4 m model and 1,000 bootstraps (112, 113). Visualizations and annotations were created using iTOL v6.7.2 (114).

Bins of interest were curated using Anvi’o v.7.1 by removing contigs with incongruent GC content and/or depth of coverage (42). Post-curation bin quality was assessed with CheckM. In addition, the CoverM v0.6.1 (115) genome command was used to calculate the abundance of each bin in its respective assembly and to estimate its percent abundance in the community, including unbinned contigs.

Genomes were annotated and screened for functional genes of interest using DRAM v0.1.2 (116), FeGenie (117), RAST-tk (118–120), METABOLIC-C (121), and BLAST (122). Putative multicopper oxidase (MCO) sequences were identified and collected using a custom python script that detected conserved MCO motifs as defined by Gräff *et al*. (62). This generated a list of potential MCOs, and these sequences were clustered at 50% identity using UCLUST (123). Clusters were identified as MCOs using BLAST. Amino acid identity (AAI) was analyzed using EzAAI v1.2.2 (124), and the results were verified against results from the Kostas lab AAI calculator (125).

### Metatranscriptomics

RNA was extracted from *in situ* frozen iron mat samples from sites ISCOA1K, DE1, and MN3 using the Qiagen RNA PowerSoil Total RNA Isolation Kit according to the manufacturer’s instructions. Fragment analysis was performed at the University of Delaware Sequencing and Genotyping Center using an Agilent fragment analyzer. All meta-transcriptome samples were sent to the DOE Joint Genome Institute (JGI) for library preparation and sequencing. An Illumina Low Input (RNA) library was constructed and sequenced 2 × 151 using the Illumina NovaSeq platform. Raw reads were processed with BBDuk (version 39.01) (126) to remove contaminants, trim adapter sequences, remove homopolymer tails with ≥5 Gs, and right quality trim reads where quality drops to 0. BBDuk was also used to remove reads that contained one or more 'N' bases, had an average quality score below 10, or had a minimum length <51 bp. Reads mapped with BBMap to contaminants (human, cat, dog, mouse, common microbial contaminants, ribosomal RNA, or known spike-ins) were also removed. Final, quality-controlled metatranscriptome reads were mapped back to their respective metagenome assemblies using Bowtie2 v.2.1.0 (127). Anvio v.8 was used to combine functional annotations from Anvio hmm sources, COG, pfam, kofam, CAZy, and FeGenie with gene calls for each metagenome assembly. R packages (readr v2.1.4; dplyr v1.1.4) were used to combine Anvio read counts and functional annotations to calculate TPM counts for each gene call and to quantify the percent rank of expressed genes (read counts > 0) (128–136).

### Metabolic model generation

Metabolic models for *Leptothrix ochracea* MAG DE1.021 were generated using ModelSEED and the KBase toolkit (137, 138). Binned genomes were annotated in KBase using RASTtk (118–120), and metabolic models were generated in the Build Metabolic Model app (v2.0.0) using the automatic Gram-negative template for reconstruction and aerobic pyruvate media for ATP gapfilling. Initial models were built using complete media, which can incorporate all compounds in the ModelSEED database. Subsequent models were built using modified glucose minimal media with Fe^2+^ and a single carbon source with the default constraints on uptake flux (glucose, mannose, formate, succinate, fumarate, maltohexaose, glycolate, acetate, and lactate; Table S10) The ModelSEED biochemistry database (v.2.6.1) was used to generate and gapfill metabolic models (137). Manual curation of these models was performed by manual addition of reactions associated with iron oxidation and electron transport in *Sideroxydans lithotrophicus* ES-1 and *Mariprofundus ferrooxidans* (49, 51), and flux through these reactions was set at a minimum of 0.1. Flux through biomass generation and reactions of interest were quantified using Flux balance analysis (v2.0.0).

## Data Availability

The data that support the findings of this study are publicly available in the NCBI and IMG. Metagenome reads, metagenome-assembled genomes, 16S rRNA amplicon sequences, and metatranscriptome reads are available under NCBI BioProject PRJNA1117470. Metatranscriptome reads are also available in IMG (IMG Genome IDs: ISCOA1K: 3300063950; MN3: 3300063952; DE1: 3300067170).

## SUPPLEMENTAL MATERIAL

Figure S1 aem.00599-24-s0001.pdf Confocal images from Spruce Point mat showing sheaths and sheath-forming cells.

Supplemental tables aem.00599-24-s0002.xlsx Tables S1 to S9.

ASM does not own the copyrights to Supplemental Material that may be linked to, or accessed through, an article. The authors have granted ASM a non-exclusive, world-wide license to publish the Supplemental Material files. Please contact the corresponding author directly for reuse.
