# Correction for Tothero et al., “*Leptothrix ochracea* genomes reveal potential for mixotrophic growth on Fe(II) and organic carbon”

**DOI:** 10.1128/aem.02044-24

**Published:** 2024-11-27

**Authors:** Gracee K. Tothero, Rene L. Hoover, Ibrahim F. Farag, Daniel I. Kaplan, Pamela Weisenhorn, David Emerson, Clara S. Chan

## AUTHOR CORRECTION

Volume 90, no. 9, e00599-24, 2024, https://doi.org/10.1128/aem.00599-24. Page 23: The following statement should be added to Acknowledgments. “We acknowledge Danielle Rushworth for performing the ICP-MS sample preparation and analysis.”

