# Supplementary material for: Leptothrix ochracea genomes reveal potential for mixotrophic growth on Fe(II) and organic carbon
Source: Appl Environ Microbiol. 2024 Aug 12;90(9):e00599-24. doi: 10.1128/aem.00599-24 (PMC11412304; doi:10.1128/aem.00599-24)
Supplement: Figure S1 — Confocal images from Spruce Point mat showing sheaths and sheath-forming cells. [file aem.00599-24-s0001.pdf]

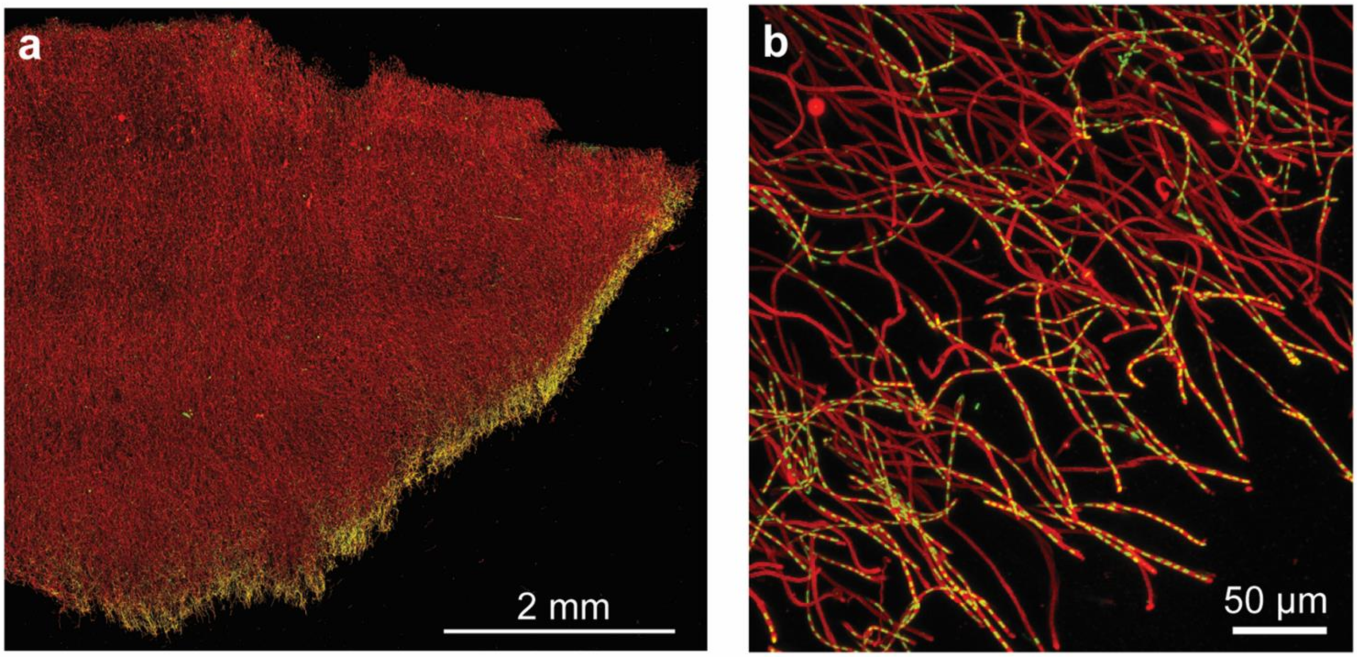

Figure S1. Confocal images from Spruce Point mat showing sheaths and sheath-forming cells. Cells are shown in green (SYTO) Fe oxyhydroxides are shown in red (rhodamine-conjugated SBA lectin) (A) View of intact mat showing abundant empty sheaths and cell growth concentrated at the edge of mat growth. (B) Confocal image showing cells surrounded by the sheath at the ends of filaments. Images from Chan et al., 2016.

Chan, C. S., McAllister, S. M., Leavitt, A. H., Glazer, B. T., Krepski, S. T., & Emerson, D. (2016). The Architecture of Iron Microbial Mats Reflects the Adaptation of Chemolithotrophic Iron Oxidation in Freshwater and Marine Environments. Frontiers in Microbiology, 7, 796. https://doi.org/10.3389/fmicb.2016.00796
